# The concepts of representation and information in explanatory theories of human behavior

**DOI:** 10.3389/fpsyg.2014.01034

**Published:** 2014-09-16

**Authors:** Renato T. Ramos

**Affiliations:** Laboratory of Psychophysiology and Neurophysiology (LIM-23), Department of Psychiatry, Institute of Psychiatry, University of São Paulo Medical SchoolSão Paulo, Brazil

**Keywords:** information, mental representation, human behavior

## Abstract

Focusing in experimental study of human behavior, this article discusses the concepts of information and mental representation aiming the integration of their biological, computational, and semantic aspects. Assuming that the objective of any communication process is ultimately to modify the receiver’s state, the term correlational information is proposed as a measure of how changes occurring in external world correlate with changes occurring inside an individual. Mental representations are conceptualized as a special case of information processing in which correlational information is received, recorded, but also modified by a complex emergent process of associating new elements. In humans, the acquisition of information and creation of mental representations occurs in a two-step process. First, a sufficiently complex brain structure is necessary to establishing internal states capable to co-vary with external events. Second, the validity or meaning of these representations must be gradually achieved by confronting them with the environment. This contextualization can be considered as part of the process of ascribing meaning to information and representations. The hypothesis introduced here is that the sophisticated psychological constructs classically associated with the concept of mental representation are essentially of the same nature of simple interactive behaviors. The capacity of generating elaborated mental phenomena like beliefs and desires emerges gradually during evolution and, in a given individual, by learning and social interaction.

## INTRODUCTION

The construction of comprehensive explanatory models of human behavior requires constant review and improvement of concepts in order to integrate different types of structures and levels of implementation. In this sense, this article discuss two concepts frequently used for modeling different aspects of human behavior in biological, psychological, philosophical, physical, and computational explanatory theories. They are the concepts of information and representation. The objective is to discuss the interdependency between both constructs with special attention to their use in experimental investigations of cognitive phenomena.

Briefly, the idea of representation discussed here is related to the brain’s capacity of developing inner states, in the form of relatively stable patterns of neuronal activity, that keep some kind of relationship with events occurring in external world. In many cases, these representations start by simple reactions to external stimuli but, due to brain’s organizational characteristics, evolve by incorporating many other elements than those directly apprehensible from the direct contact with the environment. This capacity of constructing complex mental representations results from a long evolutionary process but its basic constituents can be identified in the neuronal activity of simpler organisms in the form of reactive or conditioned behaviors, for example.

The concept of mental representation in cognitive sciences is frequently associated to complex phenomena such as beliefs and desires. This class of models, also known as representational theories of mind (RTM), consider that these states have “intentionality” in the sense that they are *about* or *refer to* things, and may be evaluated with respect to properties like consistency, truth, appropriateness, and accuracy (Cummins, 1989).

This article proposes that the general idea of intentionality or the propriety of mental states of maintaining a correlation with external events can be generalized to describe even the early stages of information processing in the nervous system. This mechanism of co-variation, in association with memory resources and the capacity of generating brain states related to abstract elements of world (more specifically the capacity of deduce the rules governing the behavior of external elements) allow the emergence of the characteristically human cognitive traits.

This broad idea of intentionality is based in a peculiar concept of information as a linking element between brains and world. Information, as used in neurobiological research, can be described as something intrinsically linked to the construction of representations but at the same time as a concept not exclusive of mental instance. Information seems to exist in natural world and human mind has a very special capability of extracting, processing, and using it to increase its capacity of interaction with the environment.

Although frequently studied separately, the concepts of information and representation can be described as having computational and semantic aspects. The term computational refers to the possibility of codification, quantification, manipulation, and physical implementation of information and representations while the term semantic refers to the meaning of both concepts in different contexts.

Information and representation will be discussed here from a neurobiological point of view but with the intention of maintaining coherence with their conceptualization in computational or artificial models of cognition. This coherence requires considering mental representations as biological phenomena, proper but not exclusive of human minds, which construction is achieved by a mechanism of information exchange with the external world. As we shall see below, although representations can be localized in the brain, their meaning does not reside exclusively in the neurobiological instance being a characteristic of the dynamic interaction between brains and environment.

In the following sections, the concepts of mental representation and information will be discussed with a declared bias toward its application in empirical problems of cognitive neurosciences. The interest in these concepts, however, is not restrict to the study of human cognition. Comprehensive discussions about classic information theory can be found in Shannon (1948), Karnani et al. (2009), Wang and Shen (2011), and Adami (2012). The nature of mental representations in philosophy, psychology, and neurosciences is discussed by Cummins (1989, 1996), Stich (1992), and Fodor (2000). Comprehensive discussions about semantic information are found in Floridi (2005), Karnani et al. (2009), Jensen et al. (2013), and Vakarelov (2014).

## THE EMPIRICAL STUDY OF HUMAN BEHAVIOR

The paradigmatic situation faced by neuroscientists during their experimental work can be described as follows: consider an individual observing an object and/or carrying out a mental task while his/her brain activity is recorded by a functional neuroimaging machine. Based in the machine’s outputs, the scientist controlling the experimental setup wants to know how the individual’s cognition works and to what extent the machine output reflects the individual experience of thinking.

Although it is possible to get some kind of information from the machine, the descriptive capacity of this paradigm is limited, especially in relation to the apprehensibility of subjective experiences. This limitation can be expressed by the qualia argument: although the scientist can know something about the individual’s internal state it is impossible for an external observer to have access to the very nature of mental processes because they involve a special quality of conscious experience that cannot be reduced to a linguistically mediated set of descriptive elements (Kanai and Tsuchiya, 2012; Ramos, 2012).

This problem can be partially reduced by questioning the individual about her/his subjective experience and checking the accuracy of her/his representations of the external world. This method, however, is limited by the capacity of the individual in accessing their own internal states. The extra-conscious character of many brain activities makes it impossible for someone to be aware and report all elements composing the cognitive experience. Even simple activities are subject to uncontrollable perceptive distortions (optical illusions, for example), spontaneous evocation of memory contents, and subtle affective states that are not consciously perceived.

Although neuroimaging techniques do not account for the qualia question, they are continually improving their capacity of detecting details of brain function in terms of anatomic location and time course of events. The information obtained by functional imaging machines is expressed in terms of electrical signals or measures of metabolic activity which must be articulated with the individual’s linguistic descriptions. Machine recordings allow the spatial localization of structures working at a given moment as well as mapping the time dynamics of their interaction (Nunez et al., 2014). Thus, functional data are collected and analyzed based in a general conceptualization of the brain as an information processing device constituted by specialized and widely interconnected substructures working in constant communication.

## INFORMATION BASED ON RECEIVER

**FIGURE 1:**
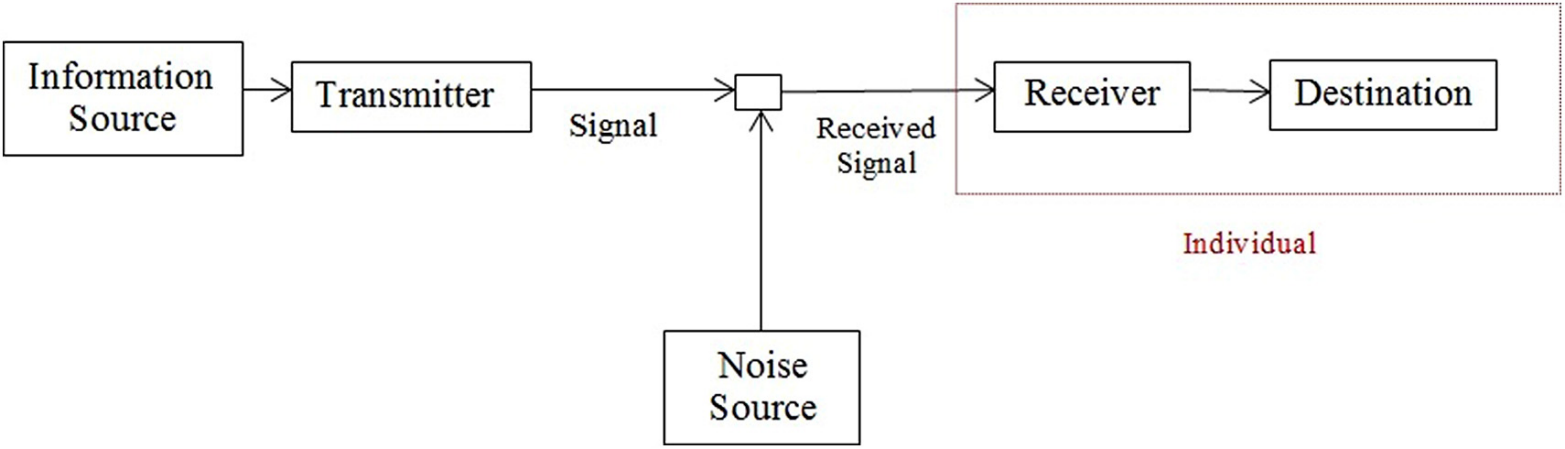
**Information system proposed by Shannon.** The red square delimits the receiver component of the system where the information’s semantic value is determined.

Probably, the most influential theory of information is that proposed by Shannon (1948) based in the concept of entropy or the uncertainty associated to the occurrence of a message. The general communication system proposed by Shannon is shown in **Figure 1**.

In a simplified form, this definition is based on the probability of occurrence of a given message among other possible ones. Although widely explored in computer sciences as well as in the study of interactions between neurons and cortical areas (Bezzi, 2007; Ward and Mazaheri, 2008), this approach is not suitable for many other applications in neurosciences. An accurate estimation of the message probability requires previous knowledge of how many other possible messages can possibly occur, which is frequently inaccessible in behavioral studies. In addition, Shannon’s model explicitly does not take in consideration the meaning of the message emitted, transmitted, and received.

The question of meaning of information, centrally important in neurosciences, has been discussed under the general topic of semantic information. Despite the lack of consensus about its definition, semantic information can be described as the data and its meaning, including or not the conditions of truthfulness (Vakarelov, 2014). The study of semantic information has focused in a number of problems, most of them systematized by Floridi (2005). The main question related to the semantic aspect of information of particular interest for this discussion is “how can data acquire their meaning” (Floridi, 2005; Vakarelov, 2010). Vakarelov (2010), for example, suggests a “pragmatic approach to information where one defines the notion of information system as a special kind of purposeful system emerging within the underlying dynamics of the world, and defines semantic information as the currency of the system. In this way, systems operating with semantic information can be viewed as patterns in organized systems.”

Returning to the general framework of Shannon’s communication system, one can says that the information transmission process is not dependent on the meaning of the message only until reaching the receiver component of the communication system. It occurs because the objective of sending a message is ultimately to provoke *changes* in receiver’s state. These changes are what determine the existence of the message from the receiver’s point of view. For example, let’s consider an individual in a dark cave populated by bats. In the absence of light and without the capacity of perceiving ultrasounds, the individual can construct only a very partial representation of the cave environment. He/she cannot determine how many bats are inside the cave, what they are doing, and if they are communicating with each other. The observer’s state cannot be modified by the events occurring in the cave due to the absence of adequate sensory mechanisms. For the bats, however, the same environment is full of meaningful information due to their capacity of emitting high frequency sounds and analyzing its echoes. If this individual is a scientist interested in understanding bat behavior, he/she can develop instruments to detect ultrasounds otherwise unperceivable and “extract” more information from the environment. Even with this new instrument, the “meaning” of this new information is not immediately clear. The only way to construct a comprehensible picture of bat activities is by establishing correlations between observable behaviors and the signals obtained by the machine. Although it is impossible for the scientist to get full access to the bat’s mind and to know how is to be like a bat, he/she can map the modifications observed in the environment and compare them with the modifications occurring in the machine states. If the machine is sufficiently precise and the bat’s behavior is sufficiently sophisticated, the scientist can build a limited map of bat’s mind.

This example can be extended to the neuroimaging techniques in general. In brain functional studies the strategy of simply correlating stationary brain states with static external stimuli has been proved meaningless. The simple mapping of all neurons firing at the moment that a specific stimulus is presented does not guarantee that the neural activity observed is related to that act of observation. In order to determine the correlation level between external world and internal brain activity, the strategy is to induce changes in object’s characteristics and observe the resulting changes occurring in brain activity. In functional brain techniques, co-varying patterns of brain activities and object presentation are usually obtained through several repetitions of stereotyped tasks which results are submitted statistical analysis. In fact, the term stimulus used in biological research can be defined as any modification of the environment that interferes with the organism’s state. In this situation, the scientist can check if the observer’s brain is receiving information by identifying changes in neural activity that correlate with changes occurring in the external world.

Therefore, the process that defines the information as something significant occurs in the *receiver* component of the system (the red box in **Figure 1**). It does not mean that other components are not relevant but the hypothesis to be explored in the next sections is that the meaning of message emerges in the receiver and any other stimuli running through the information system that is not recognized or that does not induces modifications in the receiver’s state is not information.

The Shannon communication system model has been applied in modeling each step of the nervous system’s functioning. External stimuli work as an information source to sensory cells that generate action potentials and excite the next neuron in the pathway. Cortical areas work as transmitters and receivers in relation to other areas and one person can also be modeled as transmitter, receiver, noise source, or information media according to the interest of the model. Thus, the limits of each component of an information system in an organism are arbitrary and the same formalism used to describe the interaction between two neurons can, in principle, be applied to describe the interactions between neuron nuclei or even between individuals in social interaction.

## DEVELOPING REPRESENTATIONS

The co-variation of an observer’s neural/mental states with changes occurring in the external world is the first condition for establishing a representation of objects. Many forms of representation can be generated by this process and several of them may be incomplete or inaccurate. The construction of a set of valid and useful representations requires a complementary mechanism of validation and improvement that, in biological organisms, can be implemented by the process of natural selection.

Tononi (2008) suggests that “through natural selection, epigenesis, and learning, informational relationships in the world mold informational relationships within the main complex that “resonate” best on a commensurate spatial and temporal scale. Moreover, over time these relationships will be shaped by an organism’s values, to refiect relevance for survival. This process can be envisioned as the experiential analog of natural selection. As is well known, selective processes act on organisms through differential survival to modify gene frequencies (genotype), which in turn leads to the evolution of certain body forms and behaviors (extrinsic phenotype).”

Thus, the acquisition of information and creation of mental representations occurs in a two-step process. First, a sufficiently complex brain structure is necessary to establishing internal states capable to co-vary with external events. Second, the validity of these representations must be gradually achieved by confronting them with the environment. The hypothesis discussed here is that the sophisticated psychological constructs classically associated with the concept of mental representation start from simple interactive behaviors. The capacity of using language and interacting in social groups allowed the gradual emergence of more complex human mental phenomena. This development can had occurred even by a relatively disorganized process of creation, modification, and correction of internal states in function of new inputs from external world.

Therefore, it is possible to admit that the mechanisms by which human cognition had developed are present in other classes of organisms. For example, an insect survives in its natural habitat because it can maintain a sufficiently accurate representation of external world. This representation-mediated “world-insect relationship” is limited and it even may not be considered as of cognitive nature. However, the quality and precision of this representation is the optimized result of a compromise between anatomo-physiological constraints and the necessity of providing information processing resources in the context of selective pressure in a specific niche. Partial representations may be suited to improve survival chances because they are easier to be created and corrected and faster to be implemented in natural life situations.

### REPRESENTING RULES

Another representational strategy that emerged along the evolution is the representation of the rules or patterns governing what happen in the external world. For example, conditioned behaviors in several animal species can be understood as a representation of external regularities. The increased dog’s salivation after a conditioned stimulus related to food is mediated by a representation, established by learning, of a rule of correlation between two events.

In the human brain, similar mechanisms seem to work even in more complex activities. Noelle (2012) reviewed evidences that rule-guided behaviors in humans are associated with the functioning of the prefrontal cortex, the basal ganglia, and related brain structures. The author suggests that a “dopamine-based gating mechanism interacts with standard models of synaptic plasticity to support the development of appropriately isolated and dimensional prefrontal representations, giving rise to improved generalization to novel situations when adequately diverse training experiences are provided.” According to this proposal, some regions of the prefrontal cortex may encode references or “pointers” to other prefrontal areas in a representational scheme that would allow for essentially combinatoric generalization to novel rules. This capacity of combinatoric generalization does not imply a “mere implementation” of symbolic rule-interpretation mechanisms. For Noelle, “complex interactions between the rule representations actively maintained in prefrontal cortex and the dynamic processes of more posterior neural circuits give rise to graded and context-sensitive patterns of performance that escape description by a purely symbolic rule account. Also, statistical regularities in the experiences present during the development of prefrontal cortex can profoundly shape the kinds the explicit rules that can robustly be represented and applied.”

The process of information processing based on representation of rules can be further enhanced by the creation of subsets of *a priori* representations available for use in natural situations. Innate behaviors, related to threat detection for example, require the pre-existence of relatively complex representations capable of enhancing fast protective actions. This characteristic is called preparedness of fear and phobias and it has been identified also in human behavior. Mineka and Ohman (2002) present evidences for the existence of an evolved module for fear elicitation and fear learning with four primary characteristics: “First, it is preferentially activated by stimuli related to survival threats in evolutionary history. Thus, fear-relevant stimuli lead to superior conditioning of aversive associations compared with fear-irrelevant stimuli. Second, the module is automatically activated by fear-relevant stimuli, meaning that fear activation occurs before conscious cognitive analysis of the stimulus can occur. Third, the fear module is relatively impenetrable to conscious cognitive control, and fear conditioning with fear-relevant stimuli can occur even with subliminal conditioned stimuli. Fourth, the amygdala seems to be the central brain area dedicated to the fear module.”

The high velocity required by the process of identifying threats and implementing adequate responses imply in an increased probability of errors related to the simplification of external situations, misinterpretation of new events, and ultimately the creation of distorted representations. This style of cognitive functioning can be understood under a biological perspective where, in natural situations, errors of commission (wrongly reacting to a non-threat) are more acceptable than errors of omission (not reacting to a real threat).

Other cognitive capacities like empathy and face recognizing also seem to be implemented by similar mechanisms of working with pre-prepared representations (Regenbogen et al., 2012; Kryklywy et al., 2013; Prochnow et al., 2013). Admitting that the same design strategy is used in the implementation of other cognitive functions, this mechanism of simplifying representations in order to facilitate stimuli responses may be hypothesized as playing a role in complex phenomena associated to partial or biased evaluations of external situations like folk psychological explanations and the occurrence of preconceptions in social contexts.

## CORRELATION AND INFORMATION

In order to differentiate from Shannon’s informational entropy, the term *correlational information* is proposed here, not as a measure of probability but as a measure of how changes occurring in external world correlate with changes occurring inside an agent. This concept does not depend either on the physical, biological, or linguistic nature of external object nor on the cognitive capacity of the receiver. Correlational information depends on the receiver capacity of modifying aspects of its internal states in function of changes occurring in the external environment. This receiver’s plasticity needs not to reflect every characteristic of external objects because even partial representations can be sufficient for adequate interactions with the environment. This strategy of adopting an information model based in correlations aims to emphasize the importance of the receiver component of in the general model of information system.

**FIGURE 2:**
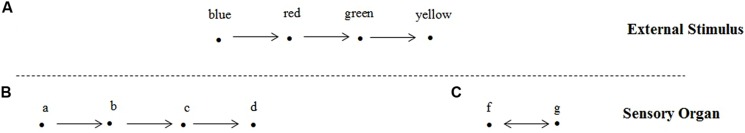
**This figure illustrates different representational capacities of sensory organs. (A)** represents a light source emitting different colors. The sensory organ illustrated in **(B)** is capable of associating the series of different states a,b,c,d where each state is related to a different color blue, red, green, and yellow. **(C)** illustrate another kind of sensory organ not capable of distinguishing blue from green and red from yellow and, therefore, representing the changes occurring in the exterior world by simplified set of states (f,g).

This approach can be illustrated as follow: let’s consider an animal visually perceiving a light source emitting different colors (**Figure 2A**). If its sensory organ has the capacity of having its state modified in a given way by each color which induces one corresponding change of state (**Figure 2B**), one can says that this animal is capable of having accurate color perception. Note that, in this model, how exactly this correspondence is physically implemented is not important. The central point is that the path blue, red, green, yellow in the external world correspond to the path a,b,c,d inside the organism. In contrast, if the sensory organ is not capable of distinguishing blue from green and red from yellow, for example, its internal representation is given by a simpler path (fig) in **Figure 2C**.

The representation expressed in **Figure 2C** is partial in comparison to that expressed by **Figure 2B** but its physical implementation by a simpler sensory organ demands less resources. If both representations have the same efficiency in preserving the animal’s life (detecting food or predators, for example), the simplest alternative may be the most advantageous unless new changes occur in the environment making the exact color perception an essential trait for survival.

According this model, the flux of correlational information along nervous system is the set of modifications gradually established along sensory cells, nerves, interneurons, and brain structures involved in behavior expression. An advantage of this concept is that these modifications are potentially detectable by functional techniques although not always accessible to an individual’s consciousness. In experimental context, even physiological manifestations like, for example changes in autonomic functioning or postural control can be considered as part of the set of information that composes mental representations. The inclusion of these not purely cognitive elements is essential, for example, in the study of emotions where several experiential elements cannot be adequately described by language.

This proposal does not imply in denying the existence of internally generated states. Although mental events can occur with a degree of independence from external influences (for example, reflections, interpretations, and mathematical thinking) the basic neural components that allowed the development of these sophisticated capacities are closely related to those working in other relatively more simple brain activities.

The human thinking process can run with a relative independence from external inputs like in mental fantasies. The correlational model proposes is that the ability of working at this level of abstraction was acquired by the gradual improvement of the capacity of using correlational information. Once acquired, this ability allows to the individual to work with independence from direct sensorial inputs and add new elements to mental contents. Although fantasies can be generated with large degree of freedom, the awareness that these contents are internally created is given by the capacity of confronting them with external inputs.

One example of internally generated state involving pre-prepared structures closely related with external events is the mirror neurons system. Originally found in macaque monkeys, in the ventral premotor cortex, area F5 and inferior parietal lobule, this group of neurons fire when the animal sees another animal (or the experimenter) performing actions similar to those pertaining to its natural repertoire of actions. Neuroimaging and electrophysiological studies indicate that mirror neurons may serve for action recognition in monkeys as well as humans, whereas their putative role in imitation and language may be realized in human but not in monkey (Oztop et al., 2013). Although primarily of motor nature, mirror neurons have been associated with mental activities like intention understanding, emotions, empathy, and speech (Acharya and Shukla, 2012).

Another examples of mental representations based in brain-environment co-variant proprieties are those involved in the orientation and movement in the space. Land (2014) points out “that the motor system requires a representation of space that maintains a consistent relationship with objects in the outside world as the body moves within it, then this could also serve as a model of a stable outside world of which we can be conscious. A high-definition representation is not necessary, all that is required is that it provides a stable framework to which detailed information, provided by the visual pathways through the occipital and temporal lobes, can be temporarily attached.”

The creation and recording of mental representations involves the gradual recruiting of relatively distant but highly connected brain components with different time dynamics. Consequently, mental representations are not localized in specific brain regions but they gradually emerge along the entire neuronal processing. This idea is compatible with several neurobiological phenomena associated with conscious experience. Shen et al. (2013) proposed that the experience of “insight,” described as an experience related to a state of understanding, which emerges into one’s conscious awareness with sudden abruptness, involves many distributed brain regions, including the lateral prefrontal cortex, cingulate cortex, hippocampus, superior temporal gyrus, fusiform gyrus, precuneus, cuneus, insula, cerebellum, and some areas of the parietal cortex.

The ability of processing complex concepts and rules governing external events is essential to the emergence of another property of human cognitive systems that is the possibility of anticipating future events. The capacity of preview the occurrence of a given stimulus can be identified even in simple organisms exhibiting conditioned behaviors. For example, the technique of olfactory conditioning of the sting extension response has been extensively used to yield new insights into the rules and mechanisms of aversive learning in insects (Tedjakumala and Giurfa, 2013).

This simple capacity of representing rules can be improved by the development of more complex neural resources. In fact, this capacity vary from one species to other (Seed et al., 2012) and along the cognitive development of each individual (Wellman et al., 2001). Moreover, there are also evidences that this representational capacity do not depend of neuronal mechanisms but also of adequate social and cultural influences (Moriguchi, 2014).

## EMERGENCE AND COMPLEXITY

The next question, central for this discussion, is how simple mechanisms of correlation allow the emergence of complex abstractions in the human mind. A possible strategy for clarifying this point is to explore complex systems theories and its applicability at the several structural and organizational levels evolved in the genesis of human behavior.

The idea that complex patterns can spontaneously emerge from simpler components is largely discussed in natural sciences and a number of theoretical ideas have been proposed to explain their occurrence like, for example agent-based models and genetic algorithms (Caticha and Vicente, 2011; Gros, 2013).

One of these theoretical models in particular, known as self-organized criticality (SOC), has received great attention as a possible explanation for the spontaneous emergence of complex patterns both at neural and behavioral levels. The concept of SOC was proposed by Bak et al. (1987) as one of the mechanisms by which complexity arises in nature. They suggested that “dissipative dynamical systems with extended degrees of freedom can evolve toward a self-organized critical state, with spatial and temporal power-law scaling behavior.” This spatial scaling leads to self-similar “fractal” structure identifiable in many conditions.

Beggs and Plenz (2003) reported evidences of this phenomenon studying organotypic cultures from coronal slices of rat somatosensory cortex. They continuously recorded spontaneous local field potentials (LFPs) using a 60 channel multielectrode array and found that the propagation of synchronized LFPs activity was described by a power-law. The authors suggested the slope of this power-law, as well as its branching parameter, indicate the presence of SOC in these preparations. (Beggs and Plenz, 2003) found evidence that the critical branching process optimizes information transmission while preserving stability in cortical networks. Simulations showed that a branching parameter at value found in the experimental preparation optimizes information transmission in feed forward networks, while preventing runaway network excitation. The authors called this pattern “neuronal avalanches” and hypothesized that it could be a generic property of cortical networks and represent a mode of activity differing from oscillatory, synchronized, or wave-like network states.

Compatible with the ideas discussed here, the identification of such patterns of functioning seems to depend on the brain functioning in context. El Boustani et al. (2009) studied intracellular activity of 15 neurons in the primary visual cortex of the anesthetized and paralyzed cat. Each neuron was recorded while presenting four full field stimuli through the dominant eye: a drifting grating at the cell’s optimal orientation and spatial frequency, a high spatial definition dense noise, a natural image animated with a simulated eye movement sequence, and a grating animated with the same eye movement sequence. The authors found the recordings displayed power-law frequency scaling at high frequencies, with a fractional exponent dependent on the spatio-temporal statistics of the visual stimuli. They also reported that this effect was reproduced in computational models of a recurrent network. They noted “that the power-law relations found here depend on the stimulus, which means that the frequency scaling exponent does not represent a unique signature of cortical network activity, but rather reflects a measure of the dynamic interplay between the sensory evoked activity and the ongoing recurrent network activity.”

The possibility of SOC being relevant for explaining complex human behavior was explored by Ramos et al. (2011) who evaluated groups of individuals with and without mental disorders in social interaction during several weeks. Although the behavior of each individual had been very different from other participants in absolute terms, the statistical description of the different groups (individuals with depression, psychosis, mania, and normal controls) showed identical patterns of behavioral variation. In all groups, comparing the behavior of individuals with themselves, small changes of behavior were very frequent while large variations were rare. The characteristic of having the same variation pattern reproduced at different levels of human activity, suggests the presence of self-similarity (Serrano et al., 2008). The curves describing the behavior of all clinical groups and controls showed the same aspect and fitted a power-law. The authors suggested that the presence of self-similarity and power-laws is compatible with the hypothesis that humans in social interaction constitute a system exhibiting SOC.

Self-organized criticality is certainly a promising concept for integrating biological and behavioral aspects of human behavior under the same causal mechanisms but it doubtless requires more empirical investigations (Hidalgo et al., 2014).

## A BRIEF COMMENT ON THE SEMANTIC QUESTION

The last important point to be discussed here is the question of the ascription of *meaning* in informational models of cognition. This is a very problematic discussion in the literature that cannot be adequately addressed in the limited scope of this article. However, the empirical research in neurosciences demands some strategy for dealing with this problem due to the impossibility of understanding many aspects of human behavior without considering some form of justification.

A possible provisional strategy is to leave the concept of meaning momentarily aside and explore a utilitarian approach of the mental representations. In a biological perspective, the immediate utility of behaviors and mental representations is increasing individual’s survival chances in different contexts. So, although informations and representations have been defined, in this correlational approach, in function of effects observed in the receiver component, their utilitarian character must be apprehended only in the context of the entire communication system.

Naturally, the idea that human cognition was molded by evolutionary mechanisms is not new. Tononi (2008) explain this hypothesis: “Brain mechanisms, including those inside the main complex, are what they are by virtue of along evolutionary history, individual development, and learning. Evolutionary history leads to the establishment of certain species-specific traits encoded in the genome, including brains and means to interact with the environment. Development and epigenetic processes lead to an appropriate scaffold of anatomical connections. Experience then refines neural connectivity in an ongoing manner though plastic processes, leading to the idiosyncrasies of the individual “connectome” and the memories it embeds.”

The general concepts of evolution theory have been used for the explanation of several kinds of behaviors and cognitive phenomena. However, this explanatory strategy still needs to be better incorporated by empirical studies. The same attention dedicated to developing neurofunctional techniques must also be dedicated to the identification and analysis to the characteristics of the environment where the behaviors are manifested. For example, this utilitarian characteristic of informational models suggests that future developments in functional brain studies must consider the use of immersive virtual reality setups as a way of controlling the behavioral context.

## CONCLUDING REMARKS

This article aimed to address some questions about the use of the representation and information concepts in the context of experimental research in cognitive sciences. The focus in the “information based on the receiver” proposed here is justified by the interest of developing objective approaches to the study of human behavior in biological and semantic terms. This search for new conceptual approaches took the risk of being superficial in its formalism but it was proposed as a first step for the description of the different elements that contribute to the construction of mental representations.

The correlational information concept discussed here aimed to be sufficiently simple to allow a naturalization of the information concept in the sense that all interaction between physical entities can be seen as an informational phenomenon. In this model, the construction of mental representations can be seen as a special case of information processing in which correlational information is received, recorded, but also modified by a complex, emergent, and possibly stochastic process of associating new elements. The validity of these new internally generated constituent elements is granted by its continuous confrontation with new external inputs and by the selection of the most adequate representations in relation to its capacity of improving survival chances.

The hypothesis is that this basic mechanism works in all animal species but, with the improved human brain capacity, it leads to the emergence of higher order or abstract descriptive elements of external objects that allow the prediction of future events. This process is possible by the manipulation of internal states representing not only objects but also the rules governing their behavior. In this model, although the content of correlational information depend on the receiver capacity of creating internal states capable to co-vary with external events, the utility of a given information can be apprehended only by the observation of the entire communication system.

The continuous process of collecting information, creating representations, generating predictions, comparing with outcomes, and adjusting them in order to optimize their accuracy is compatible with several psychological models of learning and cognitive development. This mechanism of correlational representations is also compatible with a Bayesian conception of cognitive functioning where partial or provisional representations work as estimators of *a priori* probabilities in dealing with future events (Tenenbaum et al., 2011).

The ideas discussed here represent a first approach and naturally demand deeper investigations in relation to its theoretical and empirical implications. In theoretical terms, although theories like SOC are promising in explaining human behavior, other mathematical models also deserve attention. Caticha and Vicente (2011), for example, argue that statistical mechanics can leads to aggregated predictions which can be tested against extensive data sets with partial information about populations. The process of exchanging information and learning patterns involved in these models can elicits collective emergent properties not found in individual behaviors.

In relation to the empirical research, this discussion suggests that the integrative study of the computational and semantic elements that compose human experiences will demand significant technical and theoretical improvements. Technically, the combined register of different variables like cortical electric activity, mapping of eye movements, measures of skin galvanic conductance, and postural control obtained during carefully planned cognitive activities emulated in virtual reality environments, for example, can potentially give a deeper comprehension of the mental, affective, and motor events occurring in realistic contexts.

## Conflict of Interest Statement

The author declares that the research was conducted in the absence of any commercial or financial relationships that could be construed as a potential conflict of interest.
